# The application of chimeric free anterolateral thigh flap and muscle flap for heel ulcer reconstruction: A case series and technical refinements

**DOI:** 10.1016/j.jpra.2025.08.040

**Published:** 2025-09-08

**Authors:** Rui Yang, Wenhu Jin, Ziyang Zhang, Jianping Qi, Zairong Wei

**Affiliations:** Department of Burns and Plastic Surgery, Affiliated Hospital of Zunyi Medical University, No. 149 Dalian Road,Huichuan District, Zunyi city, Guizhou Province, 563003, P.R. China

**Keywords:** Heel ulcer reconstruction, Stereoscopic restoration, Anterolateral thigh flap, Chimeric flap, Free flap

## Abstract

**Background:**

The free anterolateral thigh flap has become a preferred option for skin defect reconstruction, particularly for heel ulcers. Due to the heel's weight-bearing role and potential bone destruction from ulcers, an anatomical-functional repair is essential for functionality restoration. This study reports long-term outcomes of a chimeric ALT fasciocutaneous flap combined with vastus lateralis muscle for calcaneal ulcer reconstruction, showing restored heel weight-bearing capacity.

**Methods:**

We retrospectively analyzed nine consecutive cases of heel ulcers from June 2020 to June 2022. All patients presented with exposed calcanei, five of whom had calcaneal infections. The nine cases underwent reconstruction using free anterolateral thigh (ALT) fasciocutaneous flaps combined with vastus lateralis muscle. The appearance of the flap and the functional recovery of the feet were assessed using the Freiburg ankle scoring system at the final follow-up visit.

**Results:**

The mean follow-up time was 12 months (range 6–18 months). The free flaps survival rate was 100 %. All patients had a successful return to ambulation. A linear scar remained in the donor site. The Freiburg score at the last follow-up was 73–95, with a mean of 88.5. According to the Freiburg scores, eight cases were good, and one case was moderate.

**Conclusion:**

After the long-term follow-up, the chimeric free anterolateral thigh flap and muscle flap may restore the weight-bearing function of the heel. It can make the patient back to work and daily activities, increasing the quality of life.

## Introduction

The primary goal of surgical repair of heel ulcers is to restore and maintain their weight-bearing function.^1^ The heel is covered by the calcaneus with the hard, friction-resistant plantar fascia and thicker stragrafttum corneum skin which densely covers the calcaneus and ankle mobility tendons. It becomes the basis for standing, walking, and locomotion.^2^ Free tissue transfers are often the preferred option for the reconstruction of complex ulcer wounds in the heel. As free tissue flaps became more sophisticated, innovations and improved techniques led to the development of flap surgery for lower extremity salvage.^3^ Some commonly used flaps, such as calf flaps or plantar medial flaps,^4^ can repair the wound very well.^5^^,^^6^ However, the base of the flap is directly connected to the calcaneus and subjected to the pressure of the hard calcaneus when walking. The skin flap sometimes fails to fill the cavity under the ulcer,^2^ and sinks into the cavity under the pressure of walking. The pressure point of the heel is the residual heel tissues around the flap, which increases the pressure of the normal residual heel and unavoidably forms a new ulcer. This report aimed to describe our experience with anatomical-functional repair of heel ulcers using free anterolateral thigh flap embedded with muscle flap grafts and to report long-term functional outcomes.

## Methods

We report nine patients treated with a free lateral femoral circumflex flap to treat denervated heel ulcers between June 2020 and June 2022. There were 8 male patients and 1 female patient in the study group; their ages ranged from 29 to 85 years old, with an average age of 64.1 years. The average wound size on the heel and calcaneal was 74.8 cm^2^ (range 27 to 120 cm^2^). All patients had calcaneal exposure, and five of them had calcaneal infection. The demographic and health characteristics of our patient group are in Table 1.

**Table 1:** Patient data.

Total patients (n)	*n* = 9
Sex	
Male (n)	8
Female (n)	1
Age (years), Mean (range)	64.1 (29–85)
Wound etiology	
Diabetic foot ulcer (n)	1
Infective ulcer (n)	7
Trauma (n)	1
Comorbidity	
Diabetes mellitus (n)	1
Secondary tuberculosis (n)	1
Osteomyelitis (n)	4
Diabetic neuropathy (n)	1
Smoking (n)	8
Hypertension (n)	1
Hypoproteinemia (n)	1
Wound location	
Weight-bearing Heel (n)	9
Exposure of the Achilles tendon or calcaneus (n)	9
Both (n)	4
Achilles tendon injury (n)	1
Wound size (cm^2^), Mean (range)	74.8 (27–120)

In this study, all nine patients underwent heel ulcer wound debridement after admission to completely remove necrotic infected tissue and install a closed negative pressure drainage device. For patients with calcaneal infection, appropriate debridement of the calcaneus is performed, and the patient should be filled with antibiotic bone cement. After 7 to 21 days, the wound surface is cleaned, and the repair surgery is performed under intubation and general anesthesia. Denervated heel ulcers were reconstructed using an anterolateral thigh fasciocutaneous flap combined with a chimeric vastus lateralis flap. The range of skin flap excision is 13cm×5cm×18cm×6.5 cm. Antibiotics were routinely used for 2 to 5 days after surgery, papaverine was used for anti-vasospasm, and low-molecular-weight dextran was used for volume expansion for 7 days—partial grilling lamp for warmth. Elevate the affected limb and stay in bed for 7 days. Monitor hemoglobin and albumin levels with prompt correction of hypoalbuminemia (target albumin >3.0 g/dL) through tailored supplementation. Perform hourly flap perfusion assessment using clinical parameters (capillary refill <2 s). Implement emergent surgical exploration within 2 h if vascular thrombosis is suspected (arterial: absent Doppler signal; venous: congestive discoloration). After the sutures were removed 14 days after the operation, he began to go to the ground and gradually perform functional exercises. Observe the survival of the skin flap.

All 9 patients were followed up for 6 to 18 months, averaging 10 months. At the final follow-up, the appearance of flap, the occurrence of ulcers, and recovery of the donor site were evaluated, and the Freiburg score^7^ was used to assess the functional recovery of the foot. Upon discharge, patients are instructed to wear therapeutic footwear for diabetes during ambulation to prevent recurrence of heel ulcers.

## Operative technique

Following admission, the patient underwent debridement of the heel ulcer wound to remove necrotic infected tissue, and a closed negative pressure drainage device was placed. In cases of calcaneal osteomyelitis, necrotic bone was excised until fresh bleeding was observed in the bone marrow cavity, and antibiotic bone cement was utilized to fill the void. The repair surgery was conducted under general anesthesia with intubation 7–21 days post-wound debridement. The surgical procedure involves the following steps: 1. Flap design: A flap is created on the opposite thigh using a portable Doppler ultrasound to identify and mark the perforating vessels on the lateral side of the thigh. The flap should be fusiform and enlarged by at least 20 %. The muscle flap is selected from the vastus lateralis muscle, with a larger volume than the heel cavity. The flap size ranges from 13 cm × 5 cm to 18 cm × 6.5 cm. 2. Composite Skin Flap Harvesting: The skin on the inner side of the flap is cut, and the flap is harvested on the fascia lata. Careful separation from the preoperative perforator marking point is done, followed by a vertical incision of the fascia lata 0.5 cm away from the perforator after identification. The interval between the vastus lateralis and rectus femoris muscles was developed to expose the main trunk of the descending branch of the lateral circumflex femoral artery and its oblique branches. Initiate the dissection by separating the perforator from the main vessel at the flap entry point. Assess the direction of the perforator and the branching pattern of the main vessel to identify if the perforator vessel arises from the descending or oblique branch. After isolating the perforator, separate it towards the distal end of the main vessel. Preserving any direct perforator of the vastus lateralis muscle is crucial, as it will serve as the vascular pedicle for the muscle flap, allowing for harvesting a specific volume of the vastus lateralis muscle. If no muscle perforator is found at the distal end of the main trunk or if the muscle perforator is small, the vastus lateralis muscle flap can be harvested along with the main vessel. Subsequently, the skin on the outer side of the flap is opened, and the composite skin flap is fully harvested. The main vessel is detached to achieve the required length of the vascular pedicle, resulting in a composite skin flap with the descending or oblique branches of the lateral circumflex femoral artery as the pedicle. The composite skin flap is then transplanted to cover the heel, with the muscle flap filling the cavity and being sutured and secured to surrounding tissues while the skin flap covers the wound. An incision is made at the medial malleolus of the heel to separate the posterior tibial artery and vein. The artery of the composite skin flap is connected to the end or side of the posterior tibial artery, while the two veins are connected to the posterior tibial vein. Management of the donor area of the composite skin flap involves partial harvest of the vastus lateralis muscle, which reduces tension and allows for direct closure of the fascia lata and suturing of the skin.

Postoperatively, antibiotics are administered for 2–5 days; papaverine is used to prevent vasospasm, and low molecular weight dextran is given for 7 days to increase blood volume. Local warming with a heat lamp is applied, the affected limb should be elevated, and complete bed rest is necessary for 7 days. Correcting anemia and hypoalbuminemia is important. Close monitoring of the blood supply to the skin flap is crucial, and immediate exploration is required if vascular occlusion is identified. After suture removal at 14 days postoperatively, gradual weight-bearing and functional exercises can commence. The survival of the skin flap must be monitored. During the final follow-up, the appearance, texture, presence of ulcers, and donor area recovery should be evaluated using the Freiburg score to assess the functional recovery of the foot. The Freiburg ankle scoring system includes the evaluation of pain (30 points), function (10 points), instability (10 points), gait capability (10 points), degree of dorsiflexion and plantar flexion (10 points for each), strength (10 points), and difference of circumferences between injured and healthy extremities (10 points). According to the total scores, the results were classified as good (78–100 points), moderate (51–77 points), and poor (50 points).

## Results

A total of nine patients were monitored over a period ranging from 6 to 18 months, with an average follow-up duration of 12 months. All skin flaps that were grafted showed successful survival. Out of the cohort, one patient encountered partial flap dehiscence around the periphery 8 days post-operation, necessitating re-suturing. The remaining 8 patients experienced primary wound healing. Upon final assessment, no significant swelling or ulceration was observed in the skin flaps, and patients exhibited normal ambulation. Linear scars were noted in the donor areas of the skin flaps. Freiburg's scores at the last follow-up ranged from 73 to 95. According to the Freiburg scores, eight cases were good, and one case was moderate. Resulting in a good rate of 88.9 %.

### Case report

#### Case 1

**Figure 1:**
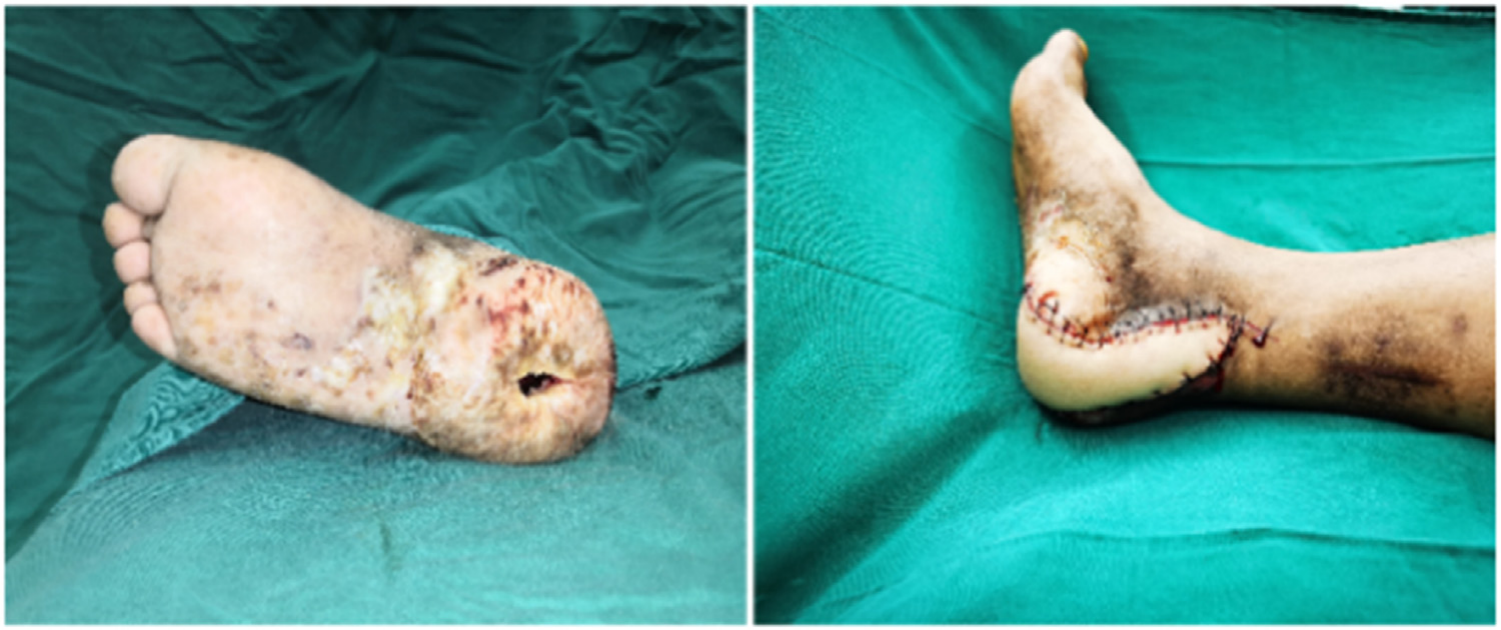
After debridement, the resultant defect was 17.5cm^2^ in size (Left) and was reconstructed by F-ALT flap with muscle component (Right).

**Figure 2:**
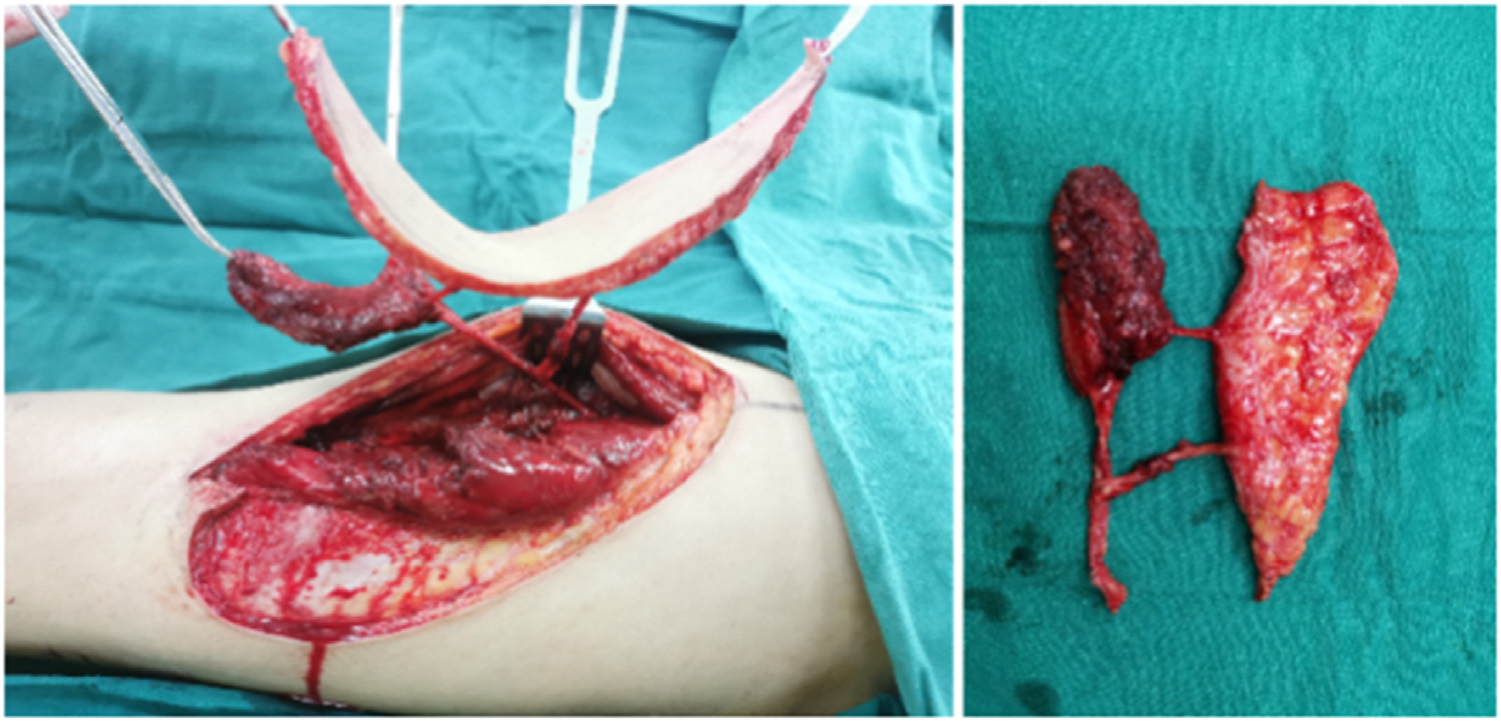
Immediate flap mobilization during surgery.

**Figure 3:**
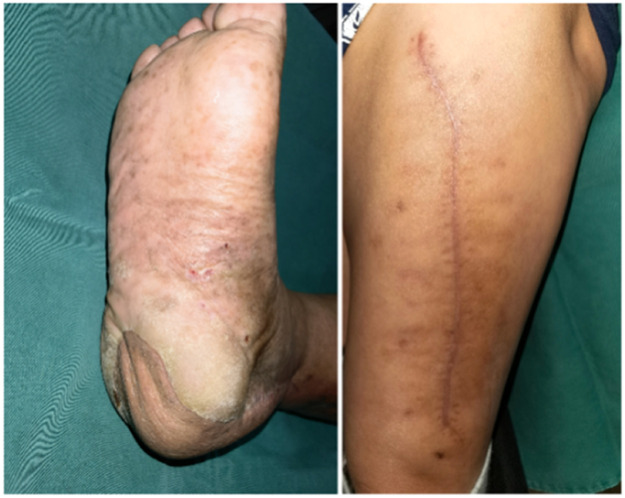
After 10 months after F-ALT flap with Muscle Component reconstruction, the patient regained full capacity for ambulation and returned to exercise (Left) and the donor area had no obvious bloating, no ulceration, no complications (Right).

A 59-year-old male presented with an ulcer and heel infection in the region of the right calcaneus. The patient underwent several extensive debridements. A muscle flap embedded in the descending branch of the left lateral femoral artery was used to reconstruct the soft-tissue defect (approximately 17.5 cm^2^). The muscle flap filled the right heel cavity and covered the wound (Figure 1). The flap artery and posterior tibial artery were severed under the microscope, and two veins were anastomosed to the posterior tibial vein, respectively (Figure 2). The postoperative flap was well viable, and at 10-month follow-up, the flap had no obvious bloating, no ulceration, no complications in the donor area, and the flap could be used for normal walking, with a Freiburg score of 86, which was good (Figure 3).

#### Case 2

**Figure 4:**
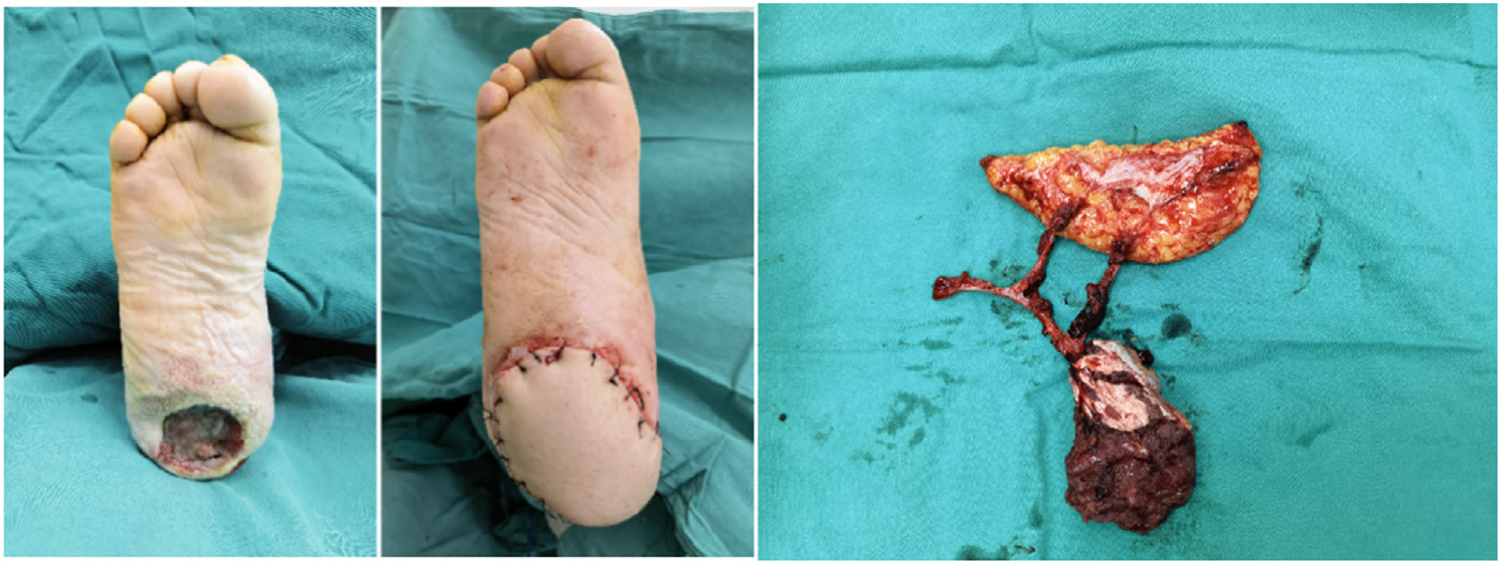
After debridement, the resultant defect was 27 cm^2^ in size (Left) and was reconstructed by F-ALT flap with muscle component (Right).

**Figure 5:**
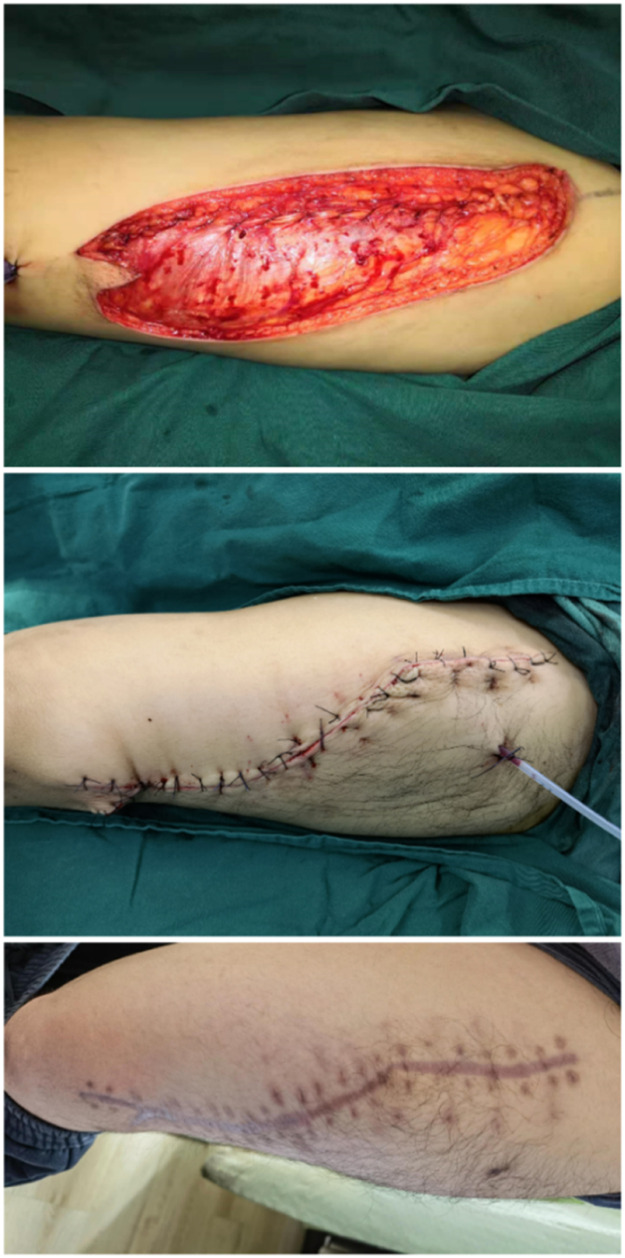
Photographs taken of the flap donor site during surgery, immediately after surgery, and 8 months postoperatively.

**Figure 6:**
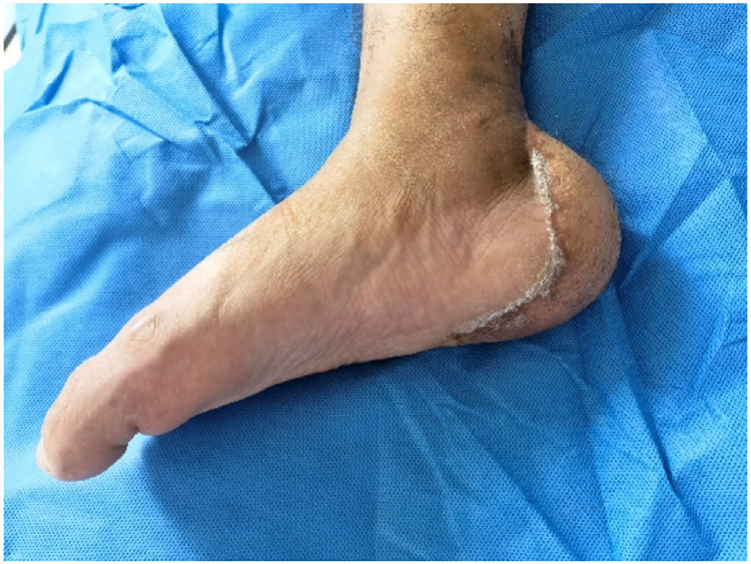
After 8 months after F-ALT Flap with Muscle Component reconstruction, the patient regained full capacity for ambulation and returned to exercise.

A 29-year-old male with a postoperative spinal fracture resulted in sensory deficits in the right foot, which led to the development of a heel ulcer. The patient underwent dilated closed negative pressure drainage of the right heel. After extensive debridement (final defect of approximately 27 cm^2^), a chimeric anterolateral thigh (ALT) flap combined with vastus lateralis muscle based on the oblique branch of the lateral circumflex femoral artery was transferred to the recipient site for reconstruction (Figure 4). The posterior tibial artery and vein were used as recipient vessels and combined end-to-end with the tibial vessels. The postoperative flap survived successfully. Follow-up after 8 months showed no significant bloat in the appearance of the flap, no ulceration, no complications in the donor area, and normal ambulation (Figure 5). The Freiburg score was 87, which was good (Figure 6).

## Discussion

As the techniques of free flap reconstruction have improved, the ultimate goal of repairing skin defects is to return to ambulation and near-normal function and minimize donor site damage.^8^ The ALT flap is our first choice of free flap for lower extremity reconstruction and comprises 53 % of our weight-bearing heel reconstruction.^9^ It has reliable anatomy.^10^ Free flap reconstruction of the weight-bearing heel can achieve high flap survival and return-to-ambulation rate.^10^ Examples included postoperative spinal and pelvic injuries or some primary peripheral nerve injuries where irreversible damage to the nerves of the lower extremities exists after treatment, leading to paraplegia in severe cases.^11^ These lower extremity nerve injuries have a small chance of returning to normal even with rehabilitation. In many cases, the restored muscle strength in the lower extremity allows for walking, but the loss of sensation or sensation leads to heel complications in the weight-bearing area.^12^ Long-standing force compression or small traumatic injuries lead to heel ulceration. The loss of nerve innervation under the slow healing of the wound. When the patients themselves find that a more serious ulcer has been formed, if not treated promptly, chronic traumatic osteomyelitis secondary to cancerous ulceration will develop, resulting in amputation.^13^ On social factors, such patients themselves can walk and have the ability to take care of themselves, Once the heel ulcer, infection limits the ability to walk and activity, aggravating the burden on the family and society. Thus, anatomical-functional repair of heel ulcers is very necessary.

Heel ulcers form over a long period, often accompanied by deep tissue destruction, creating a deep hole in the wound after the first debridement. Some commonly used flaps, such as the pedicle flap of the calf or medial plantar flap^4^ have a distinct advantage for repairing the wound. Still, the flap is directly connected to the calcaneus and subjected to the compression of the hard calcaneus when walking. Thus, the flap is prone to rupture without sensation. Moreover, because the flap sometimes fails to fill the cavity under the ulcer, it sinks into the cavity under the pressure of walking so that the pressure point of the heel is the residual heel tissue around the flap, which increases the pressure on the normal residual heel, and new ulcers are inevitably formed. Chimeric flaps are different tissue flaps for the same trunk vessel.^14^^,^^15^ The most commonly used rotary lateral femoral artery inlay flap is used clinically for many complex wounds.^16^ In this study, we used a chimeric free anterolateral thigh flap and muscle flap, in which the muscle flap was filled into the cavity to act as a cushion. The flap may be bloated in the early postoperative period. But in the late follow-up, we found that with the compression of the plantar graft flap by walking, the muscle flap volume would be significantly reduced compared with the previous one. The tissue of the flap would change, which would harden it, make it difficult to slide, and tend to converge to the tissue of the heel's skin. There is a tendency to converge to the tissue of the heel’s skin, which is distinctly different from the tissue of the lateral thigh skin. In the later stage of rehabilitation training, to strengthen the patient's self-protection awareness and wear sugar footwear,^17^ this group of patients did not develop heel ulcers after surgery.

Since the two tissues in the chimeric flap have branch-specific perfusion independence, each with a reliable blood supply. Thus, the flap placement is more flexible than the traditional muscle flap which can only cover the wound in a certain direction. The chimeric flap fills the cavity with a muscle flap to act as a cushion and repair the wound with a skin flap. They can maximize the possibility of simulating the normal heel's anatomical structure and achieving anatomical-functional repair of the wound. The chimeric flap is cut from the vastus lateralis muscle, which has little effect on the thigh and no postoperative movement disorder, and the donor site can be the primary suture.

The operator should have a certain anatomical-functional spatial thinking for the rotary lateral femoral flap embedded muscle flap intraoperative anatomical changes. There are the following precautions: ①Preoperative design should be thought of as the anatomical-functional structure of the heel, in which the cutting volume should be larger than the volume of the heel cavity, The muscle flap filling the cavity should protruding from the heel because there is a certain amount of atrophy in the postoperative walking under pressure back. The site of the skin flap design should be enlarged by at least 20 %, and designed as a shuttle (Figure 5). This ensures that the skin flap can not only cover the muscle flap but also cover the incision wound of the anastomotic vessels at the inner ankle. ② Preoperative CT angiography is required to assess the patency of the posterior tibial artery. If there is injury or atherosclerosis, an anastomosis with the anterior tibial artery anterior to the ankle should be considered. ③ Intraoperative skin flap and muscle flap are separate blood supplies. The skin flap usually carries one larger perforator vessel or two small perforator vessels. The muscle flap perforating vessels can come from the main blood vessels. it directly in the trunk-issued skin flap perforator branch distal to the trunk blood vessels and vastus lateralis muscle together with the carries, reduces the step of separating the muscle perforator branch. ④In intraoperative main vessel selection, if there is an oblique branch present, try to use the oblique branch. Because the oblique branch in the mid-upper thigh issued by the perforating branch is not only thick but also through the muscle less easy to separate, The distal main vessel of the oblique branch and the muscle are together with the excision. ⑤ The muscle flap must be fully compressed and fixed post-dissection to prevent hematoma formation.

For patients requiring thin flap reconstruction, alternatives such as the superficial circumflex iliac artery perforator (SCIP) flap or superficial gluteal artery perforator (SGAP) flap offer viable solutions. These flaps provide inherent thinness without requiring secondary thinning,^18^ and are preferred by some surgeons for distal extremity coverage. However, their use is limited by shorter pedicle length (SCIP: 3–5 cm; SGAP: 6–8 cm) compared to ALT flaps (8–12 cm), and higher susceptibility to venous congestion in high-mobility zones.^19^

We acknowledge this study's relatively small sample sizes, the lack of a comparison group, and other confounding factors that may affect functional recovery. However, its strengths include detailed intraoperative and flap data collection, a single operator, elimination of surgical reconstruction confounders, and clear postoperative follow-up.

## Conclusion

In summary, the chimeric ALT fasciocutaneous flap combined with vastus lateralis muscle demonstrates reliable blood supply anqd enables precise anatomical-functional reconstruction of denervated heel ulcers. Postoperatively, the flap exhibits a non-bulky contour, supports normal ambulation, and achieves satisfactory functional restoration of the foot.

## Ethical approval

This study was approved by the Biomedical Research Ethics Committee, Affiliated Hospital of Zunyi Medical University (XLL-2023-571) and was performed in accordance with the principles of the Declaration of Helsinki.

## Funding

Collaborative innovation center of Chinese Ministry of Education (2020–39); National Natural Science Foundation of China (82260392); Zunyi Science and Technology Plan Project Contract (HZ[2021]12).

## Declaration of competing interest

None of the authors have any conflicts of interest to disclose about the preparation and publication of this article.
